# Outlier Detection Based on Residual Histogram Preference for Geometric Multi-Model Fitting

**DOI:** 10.3390/s20113037

**Published:** 2020-05-27

**Authors:** Xi Zhao, Yun Zhang, Shoulie Xie, Qianqing Qin, Shiqian Wu, Bin Luo

**Affiliations:** 1 The State Key Laboratory of Information Engineering in Surveying, Wuhan University, Wuhan 430079, China; zhaoxi1031@whu.edu.cn (X.Z.); zhangyun198902@sina.com (Y.Z.); qqqin@whu.edu.cn (Q.Q.); 2 Institute for Infocomm Research, 1 Fusionopolis Way, #21-01 Connexis (South Tower), Singapore 138632, Singapore; slxie@i2r.a-star.edu.sg; 3 Institute of Robotics and Intelligent Systems (IRIS), Wuhan University of Science and Technology, Wuhan 430081, China; shiqian.wu@wust.edu.cn

**Keywords:** geometric multi-model fitting, residual histogram, outlier detection, alternative sampling and clustering

## Abstract

Geometric model fitting is a fundamental issue in computer vision, and the fitting accuracy is affected by outliers. In order to eliminate the impact of the outliers, the inlier threshold or scale estimator is usually adopted. However, a single inlier threshold cannot satisfy multiple models in the data, and scale estimators with a certain noise distribution model work poorly in geometric model fitting. It can be observed that the residuals of outliers are big for all true models in the data, which makes the consensus of the outliers. Based on this observation, we propose a preference analysis method based on residual histograms to study the outlier consensus for outlier detection in this paper. We have found that the outlier consensus makes the outliers gather away from the inliers on the designed residual histogram preference space, which is quite convenient to separate outliers from inliers through linkage clustering. After the outliers are detected and removed, a linkage clustering with permutation preference is introduced to segment the inliers. In addition, in order to make the linkage clustering process stable and robust, an alternative sampling and clustering framework is proposed in both the outlier detection and inlier segmentation processes. The experimental results also show that the outlier detection scheme based on residual histogram preference can detect most of the outliers in the data sets, and the fitting results are better than most of the state-of-the-art methods in geometric multi-model fitting.

## 1. Introduction

Geometric model fitting attracts a lot of attention in the computer vision field, for example, in image registration and stitching [1,2,3], structure from motion [4,5,6], visual positioning [7,8,9], object recognition [10,11,12] and 3D reconstruction [13,14]. In image registration and stitching, geometric model fitting is used to remove the outliers (wrong matches) and find a subset of reliable matches—the inliers—which conform to a given geometric model. Then these correct matches are used to estimate an accurate homography matrix. Structure from motion uses these reliable matches to recover the pose. In contrast, in visual positioning, object recognition and 3D reconstruction, geometric model fitting is used to estimate the model parameters. For example, in 3D reconstruction, robust model fitting is used to estimate multiple rigid moving objects in order to initialize multi-body structure from motion, or in the processing of 3D point clouds, where planar patches are fitted to produce intermediate geometric interpretations.

Geometric transformations are needed when two-view or multi-view images are considered, but these images often contain noise and outliers, thus a robust geometric transformation is needed to deal with these cases. It is well known that random sample consensus (RANSAC) [15] and its variants [16] are commonly used to exclude the outliers, which are single geometric model fitting methods and suitable for processing simple outliers and small noise levels. On the other hand, the geometric multi-model fitting methods extended from the single-model fitting approaches such as sequential RANSAC [17,18] and multi-RANSAC [19] usually fail when dealing with large amounts of outliers and noise. The reason is the mutual interference of these multiple models. Hence a robust multi-model fitting method should simultaneously extract multiple model instances.

So far, the most popular strategy for fitting multiple geometric models is preference analysis [20,21,22,23,24,25,26,27,28,29,30,31,32]. The multi-model fitting problem can be considered as a typical example of a chicken-and-egg problem [29]: both the data-to-model assignments and model parameters are unavailable, but given a solution of one sub-problem, the solution of the other can be easily derived. Hence, the preference analysis-based method first generates a large number of hypotheses by sampling a minimum sample set (MSS), and then performs preference analysis on the hypotheses residuals. The most classical method, J-linkage [20,21,33], adopts the binarized conceptual preference of points, which binarizes the residuals by inlier threshold, and introduces the Jaccard distance to conduct linkage clustering of the point preferences, thus the inliers are segmented into different clusters. Similar to J-linkage, T-linkage [22,24,25] uses relaxation of the binary preference function and the soft Tanimoto distance to improve the conceptual preference in J-linkage for better clustering. Unlike the common “linkage clustering with preference” strategy, robust preference analysis (RPA) [23,25] first represents the data points in the conceptual space similar to J-linkage, and then performs robust principal component analysis (PCA) and symmetric non-negative matrix factorization (NMF) to reduce the multi-model fitting problem to many single-model fitting problems, which in turn are solved with a strategy that resembles m-estimator sample consensus (MSAC) [34]. Because the outliers greatly affect the hypotheses and thus also affect the preference, both J-linkage and T-linkage get rid of outliers by means of an inlier threshold. On the other hand, kernel fitting (KF) [26] uses permutations as preferences by sorting the residuals of the hypotheses, where the Mercer kernel is built to elicit potential points belonging to a common structure and to remove the outliers. Since then permutation preference had been widely used to represent the hypotheses or the data points for model fitting [27,28,29,30,31,32]. Furthermore, instead of the random cluster models, simulated annealing (SA-RCM) [32] organizes the point preferences by permutation in a weighted graph, and the multi-model fitting task is stated as a optimization problem based on graph cut, which has been widely used for model segmentation [35,36,37,38,39] by combining the model error and smoothness of the points.

Because the fitting accuracy is seriously affected by the outliers, a series of scale estimation techniques [40,41,42,43,44] have been proposed to estimate the inlier scale and automatically eliminate the impact of outliers. Most of the scale estimator methods depend on a Gaussian distribution model: *k*-th order scale estimator (KOSE) [40] assumes that the data do not include pseudo-outliers [45] and the residuals of the inliers are Gaussian distributed; adaptive least *k*-th order scale estimator (ALKS) [40] and modified selective statistical estimator (MSSE) [41] try to find the parameter *k* in KOSE automatically; iterative *k*-th ordered scale estimator (IKOSE) [42,43] attempts to handle scale estimation with pseudo-outliers with KOSE iteratively, which is quite sensitive to the parameter *k*. However, in the geometric model fitting problem, the noise distribution is always extremely complicated, and more or less deviation is caused by using Gaussian distribution, thus the scale estimators perform poorly in geometric model fitting.

In geometric model fitting, generally the residuals of outliers to all the true models are big, which presents the notion of a consensus of the outliers that will be highlighted when the proportion of good hypotheses is big enough. However, few works have been proposed studying the outlier consensus to exclude the outliers for robust fitting. Preference analysis methods like J-linkage and T-linkage eliminate the impact of outliers by using an inlier threshold. Permutation preference doesn’t need an inlier threshold, but is very sensitive to outliers. The conceptual space in J-linkage is the binarization of the residuals by inlier threshold, which extremely compresses the residual information and decreases the differences of points belonging to the different models and thus leads to under-segmentation, while T-linkage proposes a continuous relaxation of the binary to construct the preference set for linkage clustering, which keeps the continuous residual information and the retains the differences of inliers belonging to the same model, thus it results in over-segmentation.

**Figure 1:**
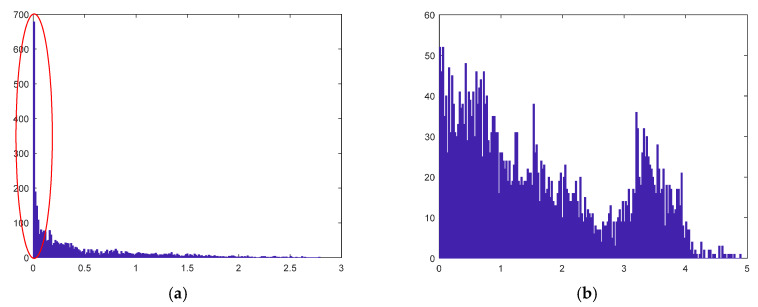
The *X*-axis represents the residual value, and the *Y*-axis represents the number of model hypotheses votes. (**a**) The residual histogram of an inlier. Peak appear in bands with small residual value, which is corresponding to the true model. (**b**) The residual histogram of an outlier. Most residual values are large and there is no obvious peak in the whole band.

The residual histograms for each data point have peaks corresponding to the true models because hypotheses generated with random sampling tend to cluster around the true model (as presented in Figure 1), which is used by residual histogram analysis (RHA) [46] to find modes in the data set. When quantizing the residuals, the peaks corresponding to the true models in histogram will be possibly transformed into one quantized value, while the flat areas corresponding to the quite different residuals will be quantized to different values. As a result, by quantifying the residual histogram, the differences of inliers belonging to the same model can be reduced and the differences of the points belonging to different models can be maintained. Especially, since the residuals of outliers to all the true models are bigger, the quantized values of outliers will tend to be bigger values corresponding to true models. In this way, if the proportion of good hypotheses close to true models is big enough, the residuals of outliers will share more common items to be bigger quantized values, which make the outliers gather away from the inliers in residual histogram preference space.

Therefore, in this paper we first introduce the residual histogram preference concept and compare its performance for outlier consensus with other different preferences including the binarized conceptual preference in J-linkage, the soft conceptual preference in T-linkage and the permutation preference. Then the residual histogram preference is applied in the proposed multi-model fitting method for detecting the outliers. After the outlier detection, an inlier segmentation process is carried out by linkage clustering, but with the permutation preference [26,30,31,47] applied on the data set with outliers removed. In order to reduce the instability of the sampling process and make the clustering process robust, an alternative sampling and clustering framework is integrated for both outlier detection process and inlier segmentation process, which involves alternately conducting random sampling within each cluster sets, and linkage clustering with the preferences obtained from the sampling hypotheses.

Hence, the contributions of this paper are three fold: (1) We propose a new preference analysis method for geometric model fitting and compare the proposed residual histogram preference with some current preferences like the binarized conceptual preference, the soft conceptual preference and the permutation preference. (2) Based on the residual histogram preference, we propose an outlier detection strategy for geometric model fitting. (3) We introduce a framework for alternative sampling and clustering in both the outlier detection and inlier segmentation for improving the stability and accuracy.

The rest of this paper is organized as follows: In Section 2, we analyze the outlier consensus in different preference spaces. In Section 3, we introduce the proposed multi-model fitting method in detail. The experiments in geometric multi-model fitting, including multi-homography matrix estimation and multi-fundamental matrix estimation, are presented in Section 4. Finally, we draw our conclusions in Section 5.

## 2. Preference Analysis on Outlier Consensus

**Figure 2:**
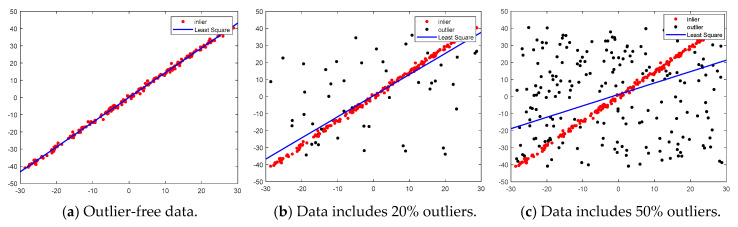
Least squares line fitting results under different degree of outlier contamination.

In multi-model fitting, outliers greatly affect the fitting accuracy, and inliers polluted by a few outliers can make the fitting parameters far from the true result. As illustrated in Figure 2, the least squares line fitting result is greatly influenced by outliers. With the increasing proportion of outliers, the bias between the fitting result and the real model also increases. It can be seen from Figure 2a that the fitted line model is very accurate when the data does not involve outliers. Therefore, in order to fit correct model instances in the presence of outliers, the outliers in the data must be removed. In fact, many geometric model fitting algorithms usually include some procedure for outlier removal.

Most of existing geometric multi-model fitting methods depend on an inlier threshold to exclude the outliers, which make the fitting accuracy greatly reliant on the inlier threshold. Because the scales of different models vary from each other, a single inlier threshold cannot satisfy all the models to exclude the outliers, thus the single inlier threshold often works poorly in geometric multi-model fitting.

Some scale estimators (KOSE, ALKS, MSSE and IKOSE) consider the distribution of inliers as a Gaussian model and try to find the inlier scale of each model in the data automatically, which successfully makes the fitting process more adaptive and robust. However, the distribution of the inliers is extremely complicated in geometric multi-model fitting, and the Gaussian model cannot fit the inlier distribution well. Therefore, Gaussian-based scale estimators work poorly in geometric multi-model fitting with non-Gaussian inlier distribution conditions.

Because outliers are not assigned to any of the true models, the residuals of outliers are bigger to all the true models, which suggests using a consensus of the outliers. When the proportion of good hypotheses is big enough after the sampling process, the residuals of outliers will share more common items to have big values. However, the consensus of the outliers is not obviously presented in the residual space. Because the scales of different models are different, the extent of the “big” values in different models is also different. On the other hand, the pseudo-outliers will tend to be much closer to the outliers in residual space, which also have many common items with big values. Currently the preference of the data points, designed from the residuals like conceptual space in J-linkage or soft conceptual space in T-linkage, can highlight the consensus of the outliers from the inliers or pseudo-outliers with the help of an inlier threshold. Permutation preference decreases the impact of the inlier scale difference by sorting the residuals and getting a serial number as the preference for data points, which also obviously represents the consensus of the outliers. Similarly, a residual histogram is quite a good way to reduce the influence of inlier scale differences by quantizing the residuals according to each model. Moreover, residual histograms can compress the residual according to the histogram, and when used to present the consensus of the outliers, the difference of outliers will be greatly compressed, thus it will make the outliers gather away from the inliers. We present the detailed preference analysis on outlier consensus for residual histogram preference below.

### 2.1. Binarized Conceptual Preference in J-Linkage

Preference analysis-based method was first used in J-linkage [20], in which the consensus set of each hypothesis is computed by collecting the points whose residual is lower than inlier threshold. In this way each point can be seen as the preference set of the hypotheses with the residuals less than inlier threshold. Given the data point set $X=\left\{x_{1},x_{2},\cdots ,x_{N}\right\}$, the hypotheses set $H=\left\{h^{1},h^{2},\cdots ,h^{j},\cdots ,h^{M}\right\}$ after the hypothesis generation, and the residual matrix $R=\left\{r^{1},r^{2},\cdots ,r^{j},\cdots ,r^{M}\right\}$, where $r^{j}={\left[r_{1,i},r_{2,j},\cdots ,r_{i,j},\cdots r_{N,j}\right]}^{T}$ refers to the residuals of hypothesis $h^{j}$ to all the data points in X, N is the data number, and M is the number of hypotheses. Then the conceptual space S in J-linkage is the binarization of residual matrix R by an inlier threshold ϑ given by:

(1) $$S_{i,j}=\{\begin{array}{cc} \begin{array}{l} 1 \end{array} & \begin{array}{l} r_{i,j}<\vartheta \end{array}\\ 0 & othersize \end{array}$$

where ϑ is the inlier threshold used to determine whether a point is inlier. Then the conceptual representation $S_{i}={\left[S_{i,1},S_{i,2},\cdots ,S_{i,j},\cdots ,S_{i,M}\right]}^{T}$ of point $x_{i}$ indicates which model point $x_{i}$ prefers, thus making the conceptual preference of point $x_{i}$. The preference set can be defined by $PS_{i}=\left\{j|S_{i,j}=1\right\}$, which represents the hypotheses set of point $x_{i}$ preference. And the Jaccard distance is proposed to measure the distance $d_{J}(i,j)$ between preference set $PS_{i}$ and $PS_{j}$:

(2) $$d_{J}(i,j)=\frac{\left|PS_{i}\cup PS_{j}\right|-\left|PS_{i}\cap PS_{j}\right|}{PS_{i}\cup PS_{j}}.$$

The conceptual space in J-linkage can be considered as ${\left\{0,1\right\}}^{H}=\left\{\phi :H\to \left\{0,1\right\}\right\}$, and points belonging to the same structure have similar conceptual representations, thus making the linkage clustering effective to segment the inliers. While for the outliers, whose residuals are bigger to all the true models, when the proportion of good hypothesis is high enough, the conceptual preference of outliers will share more common items to be zero, which will make the outliers aggregately distribute away from the inliers on conceptual preference space.

### 2.2. Soft Conceptual Preference in T-Linkage

Unlike the binarized conceptual space in J-linkage, T-linkage [22] introduces a relaxation of the binary representation by preference function, which allows the preferences of a point integrating more specific information on residuals. The preference function of point $x_{i}$ is defined by:

(3) $$\phi_{i,j}=\{\begin{array}{cc} \begin{array}{l} e^{-r_{i,j}/\tau } \end{array} & \begin{array}{l} r_{i,j}<5\tau \end{array}\\ 0 & r_{i,j}\geq 5\tau \end{array}$$

where the time constant τ plays the same role of the inlier threshold ϑ in Equation (1). Then the soft conceptual preference of point $x_{i}$ is defined by $\phi_{i}={\left[\phi_{i,1},\phi_{i,2},\cdots ,\phi_{i,j},\cdots ,\phi_{i,M}\right]}^{T}$, each element in $\phi_{i}$ represents the conversion of the residual $r_{i,j}$ through Equation (3), where $r_{i,j}$ is the residual of point $x_{i}$ to hypothesis j. The distance between two soft conceptual preference $\phi_{i}$ and $\phi_{j}$ is defined as *Tanimoto* distance as follows:

(4) $$d_{T}(i,j)=1-\frac{\langle \phi_{i},\phi_{j}\rangle }{{\left\|\phi_{i}\right\|}^{2}+{\left\|\phi_{j}\right\|}^{2}-\langle \phi_{i},\phi_{j}\rangle }$$

where the notation 〈⋅,⋅〉 indicates the standard inner product, and ‖⋅‖ is the induced norm.

### 2.3. Permutation Preference

The permutation preferences are also extracted from the hypotheses residuals [29,47]. When calculating the permutation preference of point $x_{i}$, we first sort the residuals of $x_{i}$ as $SR_{i}=\left\{r_{i,\tau_{j}^{i}}|r_{i,\tau_{1}^{i}}\leq r_{i,\tau_{2}^{i}}\leq \cdots \leq r_{i,\tau_{j}^{i}}\leq \cdots \leq r_{i,\tau_{m}^{i}}\right\}$, and then the permutation preference of point $x_{i}$ is ${\overset{⌣}{\tau }}^{i}=\left\{\tau_{j}^{i}|r_{i,\tau_{1}^{i}}\leq r_{i,\tau_{2}^{i}}\leq \cdots \leq r_{i,\tau_{j}^{i}}\leq \cdots \leq r_{i,\tau_{m}^{i}}\right\}$. In practice, the permutation preference is often used as a top-*k* list rather than a full rankings list, i.e., $\tau^{i}=[\tau_{1}^{i},\tau_{2}^{i},\cdots ,\tau_{k}^{i}]$, 1≤k≤m.

When comparing two points $x_{i}$ and $x_{j}$ with the corresponding permutation preferences $\tau^{i}$ and $\tau^{j}$, the Spearman footrule distance $d_{F}(i,j)$ [29,47] between preferences $\tau^{i}$ and $\tau^{j}$ is computed as follows:

(5) $$\begin{array}{l} d_{F}(i,j)=\sum_{\eta \in \tau^{i}\cup \tau^{j}}\left|\phi (\eta ,\tau^{i})-\phi (\eta ,\tau^{j})\right|\\ \phi (\eta ,\tau^{i})=\{\begin{array}{cc} t & if\;\eta ==\tau_{t}^{i}\\ k+1 & if\;\eta \notin \tau^{i} \end{array} \end{array}$$

where η is the union of $\tau^{i}$ and $\tau^{j}$. $\phi (\eta ,\tau^{i})$ equals t, which represents the position of an element in $\tau^{i}$ when element in η is also exist in $\tau^{i}$. Conversely, $\phi (\eta ,\tau^{i})$ equals (k+1), where k is the number of elements in $\tau^{i}$. Moreover, the preferences $\tau^{i}$ and $\tau^{j}$ are identical when $d_{F}(i,j)=1$, the preferences $\tau^{i}$ and $\tau^{j}$ are not identical when $d_{F}(i,j)=0$.

### 2.4. Residual Histogram Preference

The residual histograms for each data point have quite significant features whose peaks correspond to the true models because hypotheses generated with random sampling tend to cluster around the true model, which was used by residual histogram analysis (RHA) [46] to find modes in the data set. Usually the residual histograms of the inliers are skewed right, which is not in line with the outliers. Equivalent with RHA, we introduce a more convenient residual histogram method for representing the data points. The residual histogram preference namely conducts quantization on residuals *R*, and takes the quantized value ${\overset{⌣}{q}}_{i,j}$ for representation by:

(6) $$\begin{array}{l} {\overset{⌣}{q}}_{i,j}=\left\lceil \frac{r_{i,j}-r_{\min}^{j}}{r_{\max}^{j}-r_{\min}^{j}}*\theta \right\rceil \\ r_{\max}^{j}=\max\left\{r_{1,j},r_{2,j},\cdots ,r_{i,j},\cdots ,r_{N,j}\right\}\\ r_{\min}^{j}=\min\left\{r_{1,j},r_{2,j},\cdots ,r_{i,j},\cdots ,r_{N,j}\right\} \end{array}$$

where θ refers to the quantization level. When using the quantized residuals to represent the hypotheses or the data points, a valid quantization length λ is needed to decrease the complexity of the residual histogram preferences. Then the quantized value ${\overset{⌣}{q}}_{i,j}$ is transformed as follows:

(7) $$q_{i,j}=\{\begin{array}{cc} \begin{array}{l} {\overset{⌣}{q}}_{i,j} \end{array} & \begin{array}{l} {\overset{⌣}{q}}_{i,j}\leq \lambda \end{array}\\ 0 & {\overset{⌣}{q}}_{i,j}>\lambda \end{array}.$$

We will introduce the selection principle on the quantization length λ in detail in Section 4. In this way, we can obtain the residual histogram matrix $Q=[q_{1}^{T},q_{2}^{T},\cdots ,q_{N}^{T}]$. The residual histogram preference for data point $x_{i}$ is the *i*th row of Q, i.e., $q_{i}=[q_{i,1},q_{i,2},\cdots ,q_{i,j},\cdots ,q_{i,M}]$. When comparing two residual histogram preferences $q_{i}$ and $q_{j}$, the distance measurement $d_{W}(i,j)$ defined by:

(8) $$\begin{array}{l} d_{W}(i,j)=\{\begin{array}{cc} \begin{array}{l} 1-\frac{\sum_{t=1}^{M}\varphi (q_{i,t},q_{j,t})}{\max(\rho (q_{i}),\rho (q_{j}))} \end{array} & \begin{array}{l} if\;\max(\rho (q_{i}),\rho (q_{j}))\neq 0 \end{array}\\ 1 & else \end{array}\\ \varphi (q_{i,t},q_{j,t})=\{\begin{array}{cc} \begin{array}{l} 1 \end{array} & \begin{array}{l} if\;q_{i,t}=q_{j,t},q_{i,t}\neq 0 \end{array}\\ 0 & else \end{array}\\ \rho (q_{i})=\sum_{t=1}^{M}\varphi (q_{i,t},q_{i,t}) \end{array}$$

where $\varphi (q_{i,t},q_{j,t})=1$ when the quantized value $q_{i,t}$ between point $x_{i}$ and hypothesis t is equal with $q_{j,t}$, conversely, $\varphi (q_{i,t},q_{j,t})=0$.

### 2.5. Comparison of the Four Preferences on Outlier Consensus

In the binarized conceptual preference in J-linkage, the residuals bigger than the inlier threshold are set to 0 and the smaller are set to 1, the residuals of outliers are bigger for all the true models, and when the proportion of the good hypotheses is high enough, the binarized conceptual preference of the outliers will have more common items to be 0 with a proper inlier threshold. When projecting to the binarized conceptual preference with the Jaccard distance, the outliers will gather together.

Similar to the binarized conceptual preference in J-linkage, the soft conceptual preference in T-linkage cuts off the residuals bigger than the inlier threshold to 0, and the soft conceptual preference of the outliers will have more common items to be 0 with a proper inlier threshold, thus the outliers gather together in the soft conceptual preference space.

As for permutation preference, the residuals of outliers are bigger for all the true models and the residuals of the inliers are small for the hypotheses close to true models, the top-*k* items of the outliers’ permutation preferences will share quite few common list with the inliers’, while outliers’ permutation preferences will more likely to have more common items with each other when using a proper permutation length, thus the outliers gather away from the inliers in permutation preference space.

Because the residuals of outliers will be bigger for all the models in the data, the bigger residuals will more likely tend to be close to λ or 0 (Equation (7)) after quantization. In this way, the residual histogram preferences of the outliers will tend to have more values with 0 or λ, and when the proportion of good hypotheses is high enough, most of the values in the residual histogram preferences of the outliers will tend to be close to λ or 0, whereas the corresponding inliers will have quite small values. When projecting the data points into residual histogram preference space with the distance measurement in Equation (8), most of the outliers will present a concentrated distribution, and will be far away from the inliers, thus the outliers are easy separated from the inliers.

**Figure 3:**
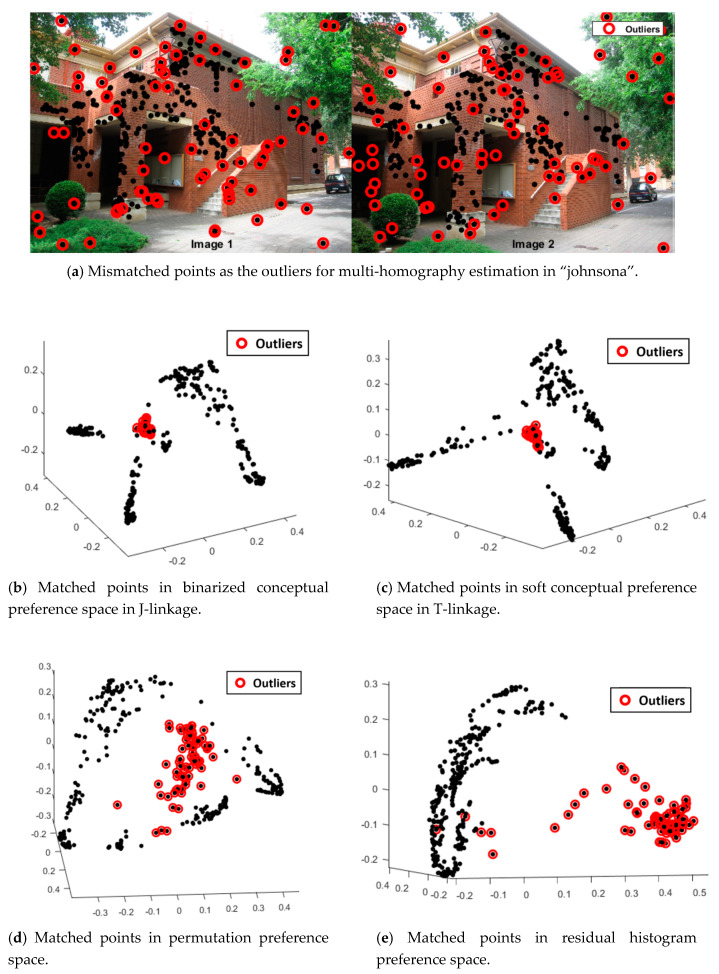
The distribution of matched points for homography estimation in different preference spaces.

As shown in Figure 3, the matched points between “Image1” and “Image2” in “johnsona” for homography estimation are presented in Figure 3a, in which the outliers (usually mismatched points) are labeled by red circle. After sampling from the matched points, a great amount of homographies can be calculated as the hypothesis models, and then the Sampson distance is used for calculating the residuals, finally the preferences of the matched points can be obtained to transform the matched points into preference space. The distribution of the matched points in binarized conceptual preference space, soft conceptual preference space, permutation preference space and residual histogram preference space are presented in Figure 3b−e, respectively, and the mismatched points as the outliers are labeled with red color corresponding to Figure 3a.

As shown in Figure 3b–e, outliers in these four preference spaces gather away from the inliers. Although outliers in binarized conceptual preference space (Figure 3b) and soft conceptual preference space (Figure 3c) are even more concentrated, both of these preferences need a proper inlier threshold, and the outlier cluster is seriously mixed with inliers, while the outlier cluster in permutation preference space (Figure 3d) seems pure, but it is more dispersed and the boundary line with the inliers are not obvious. The outliers in residual histogram preference space (Figure 3e) are quite obviously separated from the inliers, and only a few outliers are mixed in the inlier cluster, which makes the outlier detection in the residual histogram preference space quite successful and always better than the other three preference spaces.

## 3. Proposed Multi-Model Fitting

Because the outliers greatly affect the fitting accuracy, the proposed fitting method should detect the outliers first, and then conduct inlier segmentation on the data without outliers. Hence our proposed method consists of two main parts: outlier detection and inlier segmentation. As presented in Section 2, the residual histogram preference shows great superiority in presenting the outliers’ consensus, so the outlier detection will be conducted on the residual histogram preference space. In this section, we will first explain how the residual histogram preference is used to detect the outliers, and then the inlier segmentation, which segments the inliers belonging to different models, will be presented in detail.

### 3.1. Outlier Detection

**Figure 4:**
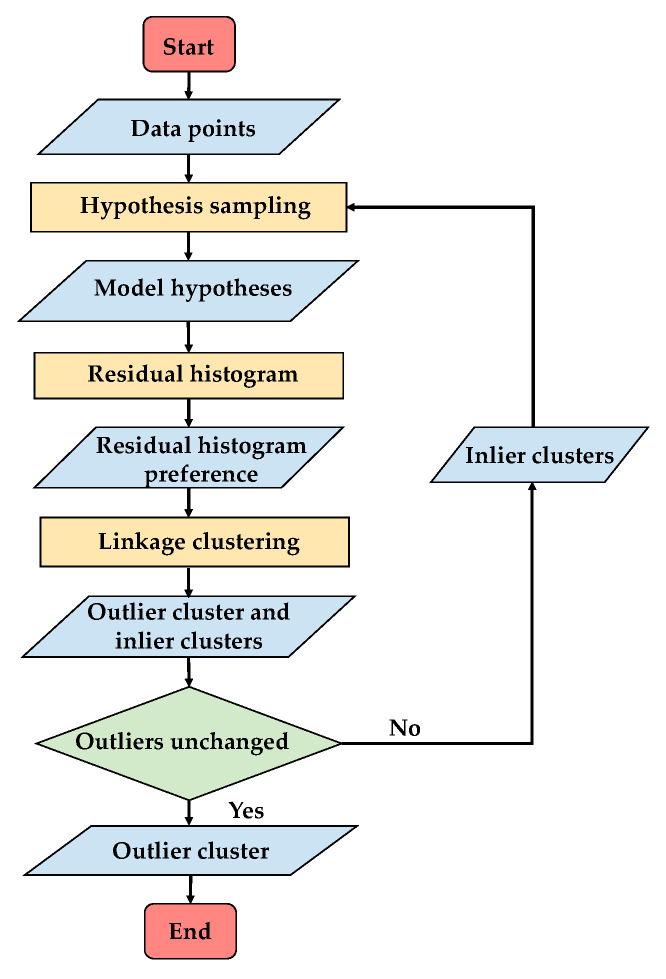
The flowchart of outlier detection.

As presented in Section 2, outlier consensus is well highlighted in residual histogram preference space, and the outliers can be well separated in the residual histogram preference space. The outlier detection is actually used to conduct outlier clustering in the residual histogram preference space. Firstly, the points are transformed into the residual histogram preference space, and then a linkage clustering method is proposed for clustering. Most of the time, the sampling process makes the result unstable, an alternative sampling and clustering framework is adopted to make the result stable by iterating the sampling and clustering processes. Also, this alternative framework can improve the sampling and clustering results. The flowchart of the proposed outlier detection is presented in Figure 4.

The proposed outlier detection method starts with a sampling process to generate enough hypotheses, and then calculates the residuals to construct the preferences for the data points. Similar to the sampling method in J-linkage [20], which gives neighboring points a higher probability to be selected, the sampling process of the proposed method enhances the spatial correlation hypothesis that neighboring points tend to be inliers for the same model, by directly selecting the MSS within a region. Firstly, the data points are divided into several sub-regions by the Euclidean distance, and we then conduct random sampling on each sub-region. In this way, inliers from the same structure will be more likely to be selected to make up the MSS and the more good hypotheses will be generated.

After the hypothesis generation, residuals for each hypothesis will then be calculated and get the residual matrix. The residual histogram preference for the data points can be obtained by method presented in Section 2.4, which will be used for linkage clustering to cluster the outliers. For the linkage cluster process, in each iteration, two points with the minimum residual histogram preference distance are merged into a cluster, and we then update the distance using the single linkage method, with the minimum distance of two elements as the distance of the two clusters. After the linkage clustering, the data points will be segmented into several clusters. Because the residual histogram preferences of the outliers will tend to have more large quantized values or a value of 0 (when the quantized value is larger than the valid quantization length λ), we can determine whether a point is an outlier by the outlier index ξ calculated as follows:

(9) $$\begin{array}{l} \xi_{i}=\frac{\sum_{j=1}^{M}{\overset{⌣}{q}}_{i,j}}{M}\\ {\overset{⌣}{q}}_{i,j}=\{\begin{array}{cc} \begin{array}{l} q_{i,j} \end{array} & \begin{array}{l} q_{i,j}\neq 0 \end{array}\\ \lambda & else \end{array} \end{array}$$

where $q_{i,j}\in q_{i}$. $q_{i}$ is the residual histogram preference of point $x_{i}$ (Equation (7)). M is the number of hypotheses. The bigger $\xi_{i}$ is, the more likely $x_{i}$ is an outlier. In order to determine whether the cluster is the outlier cluster, the average outlier index of all the points $\xi_{c^{t}}$ in the cluster t is calculated by Equation (10):

(10) $$\xi_{c^{t}}=\frac{\sum_{k=1}^{card\left\{c^{t}\right\}}\xi_{k}}{card\left\{c^{t}\right\}},\;x_{k}\in c^{t}$$

where card{·} counts the number of points in cluster t. Finally, the cluster with the maximum average outlier index is selected as the outlier cluster $c^{O}$:

(11) $$c^{O}=c^{t},\;t=\underset{j}{\arg\max}\xi_{c^{j}}$$

By the linkage clustering, most of the outliers in the data points will be detected, but because of the random sampling process, the detected outliers will change slightly every time. In order to decrease the change and further improve the outlier detection, an alternative sampling and clustering framework is proposed to conduct sampling the hypotheses and then carry out linkage clustering alternately, which make it possible to sampling the hypotheses in inlier clusters to further increase the proportion of the good hypotheses, and in turn improve the preference for better linkage clustering.

As a result, the whole process for outlier detection is summarized here. Initially, we perform the random sampling within the sub-regions, which are divided by the Euclidean space distance of the data points, and the initial outlier cluster is detected by linkage clustering with the residual histogram preference. Then, in turn, the inlier clusters obtained by the linkage clustering are regarded as sub-regions for the random sampling to generate hypotheses and, in the same way, to perform linkage clustering with the residual histogram preference to detect the outlier cluster. Thus, the sampling and clustering processes are iteratively conducted until the outlier cluster is unchanged. The details of the algorithm are listed in Algorithm 1.
**Algorithm 1** Outlier Detection 1: Divide X into m sub-regions $D=\left\{d^{1},d^{2},\cdots ,d^{m}\right\}$ by Euclidean space distance of the points, and initialize outlier cluster c^O=0;2: Conduct random sampling on each sub-region and generate hypotheses H; 3: Calculate the residual histogram preference matrix Q; 4: Calculate the distance matrix $d_{w}$ for the residual histogram preferences of points i and j; 5: Conduct linkage clustering with distance and obtain clusters $C=\left\{c^{1},c^{2},\cdots ,c^{n}\right\}$;6: Calculate the outlier index $\xi =[\xi_{c^{1}},\xi_{c^{2}},\cdots ,\xi_{c^{n}}]$ for each cluster in C, and select the cluster with the maximum outlier index as the outlier cluster $c^{O}=c^{t}$, $t=\arg\max_{j}\xi_{c^{j}}$, inlier clusters $C^{I}=C-c^{O}$;7: If $c^{O}=={\hat{c}}^{O}$, return $c^{O}$ as the outlier detection result; else ${\hat{c}}^{O}=c^{O}$, $D=C^{I}$, and return to step 2.

### 3.2. Inlier Segmentation

**Figure 5:**
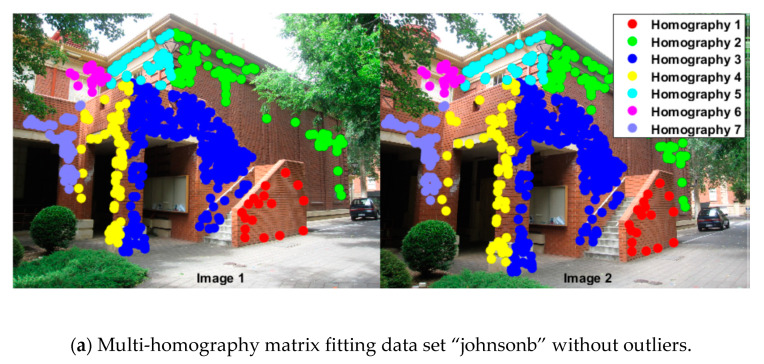

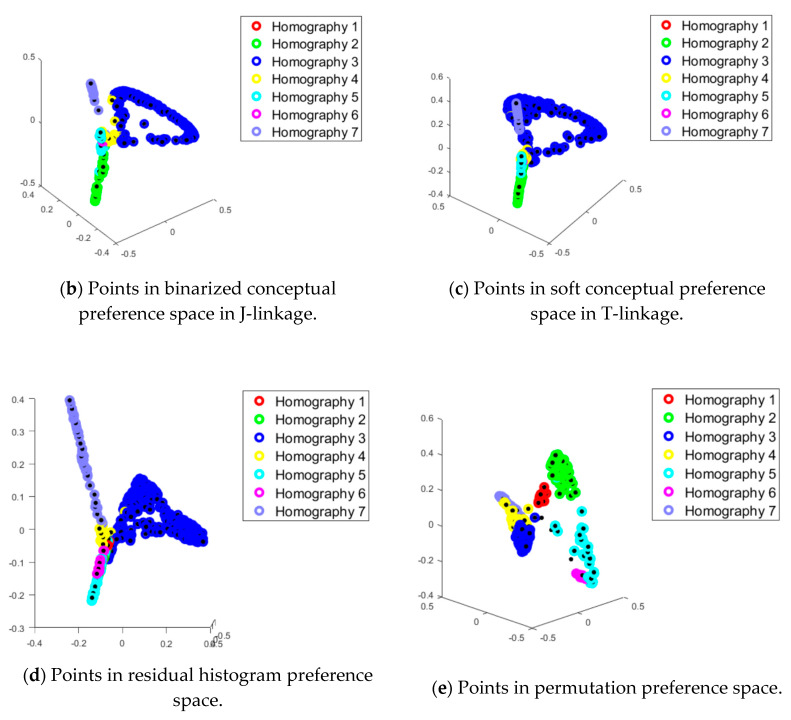
MDS (Multiple Dimensional Scaling) plots for “johnsonb” without outliers by using different preferences.

The outlier detection process usually makes it possible to detect most of the outliers in the data set, and after removing the outliers from the data set, the inlier segmentation is carried out on the remaining data set. The inlier segmentation process is conducted by using linkage clustering integrated with the alternative sampling and clustering framework, similar to the outlier detection process. However, during the linkage clustering, permutation preference [26,30,31,47] is used instead of residual histogram preference. This is because permutation preference presents a better performance in practice than residual histogram preference and conceptual preference in J-linkage or T-linkage when dealing with data with few outliers (Figure 5).

Figure 5 shows points in four different kinds of preference spaces from “johnsonb” (from the AdelaideRMF data set [28]) after outliers are removed. These preference spaces include the conceptual preference space in J-linkage and T-linkage, and the residual histogram preference space and the permutation preference space, where the data points belonging to different homography model inliers are labeled with different colors. Figure 5b–d show that inliers for the model with a large inlier scale (inliers for Homography 3, labeled with the blue circles) show a scattered distribution, but inliers for the models with a smaller inlier scale (inliers for Homography 1, 4, 6) are covered by the other model inliers, and inliers for models with different inlier scales are unevenly distributed in preference space. Meanwhile, for the permutation preference in Figure 5e, inliers for models with both a large inlier scale and a smaller scale show a concentrated distribution, and the inliers for the model with a smaller inlier scale are not seriously covered by the larger ones in permutation preference space, which is beneficial for extracting models with a smaller inlier scale.

**Figure 6:**
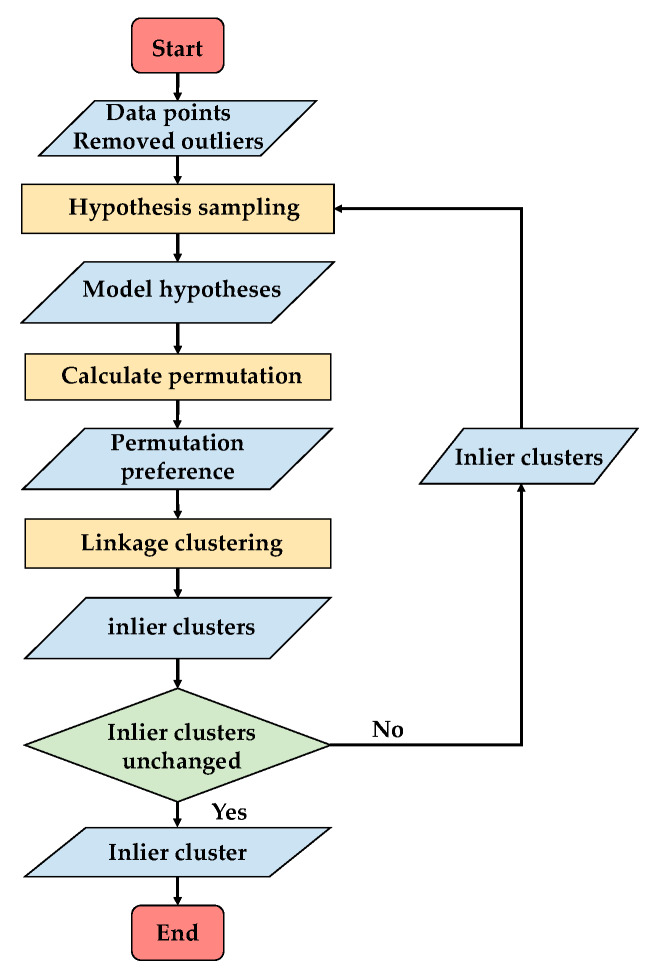
The flowchart of inlier segmentation.

The inlier segmentation follows the alternative sampling and clustering framework, as presented in Figure 6. Similar to the inlier detection process, and the number of clusters is not limited to the inliers and outliers but changed to the actual obtained clusters. In the same way, the initial hypotheses are first generated by random sampling within sub-regions $\tilde{D}=\left\{{\tilde{d}}^{1},{\tilde{d}}^{2},\cdots ,{\tilde{d}}^{\tilde{m}}\right\}$ divided with the Euclidean distance on data points ${\tilde{X}}^{I}=X-c^{O}$ with outliers removed. The permutation preference is then calculated for each point to carry out the linkage clustering and obtain the inlier clusters $\tilde{C}=\left\{{\tilde{c}}^{1},{\tilde{c}}^{2},\cdots ,{\tilde{c}}^{\tilde{n}}\right\}$. In turn, the sub-regions are updated with the inlier clusters $\tilde{D}=\left\{{\tilde{c}}^{1},{\tilde{c}}^{2},\cdots ,{\tilde{c}}^{\tilde{n}}\right\}$, and the hypotheses are generated on the updated sub-regions. This alternative sampling and clustering framework iteratively conducted until the inlier clusters are almost unchanged. The details of the algorithm are presented in Algorithm 2.
**Algorithm 2** Inlier Segmentation1: Divide ${\tilde{X}}^{I}$ into $\tilde{m}$ sub-regions $\tilde{D}=\left\{{\tilde{d}}^{1},{\tilde{d}}^{2},\cdots ,{\tilde{d}}^{\tilde{m}}\right\}$ by Euclidean space distance of the points, and initialize inlier cluster C˜O=0;2: Conduct random sampling on each sub-region and generate hypotheses $\tilde{H}$; 3: Calculate the permutation preference matrix P; 4: Calculate the Spearman footrule distance matrix F for the permutation preferences of points i and j Equation (5);5: Conduct linkage clustering with distance F and obtain clusters $\tilde{C}=\left\{{\tilde{c}}^{1},{\tilde{c}}^{2},\cdots ,{\tilde{c}}^{\tilde{n}}\right\}$6: If $\hat{C}==\tilde{C}$, return $\tilde{C}$ as the inlier segmentation result; else $\hat{C}=\tilde{C}$, $\tilde{D}=\tilde{C}$, and return to step 2.

## 4. Experiments

In this section, we describe the experiments undertaken in geometric multi-model fitting, including multi-homography estimation and multi-fundamental matrix estimation, which are fundamental issues in image stitching [48,49] and visual localization [50]. Comparisons on the segmentation accuracy for real data with some of the state-of-the-art methods (PEARL [35], SA-RCM [32], J-linkage [20], and T-linkage [22]) are made to present the characteristics of the proposed method, and the corresponding outlier detection results are also given. The reason for choosing PEARL, J-linkage, SA-RCM and T-linkage as the comparison is not only because they are all state-of-the-art methods, but also because J-linkage and T-linkage are algorithms based on preference analysis, which is suitable for a comparison with our proposed method. Both the multi-homography estimation experiment and the multi-fundamental matrix estimation experiments were based on the AdelaideRMF [28] data set. In addition to the fitting preference images, we also use the overall misclassification percentage (number of misclassified points divided by the number of points in the data set) [51] to present the fitting performance when dealing with the multi-homography and multi-fundamental matrix estimation.

### 4.1. Multi-Homography Estimation

**Figure 7:**
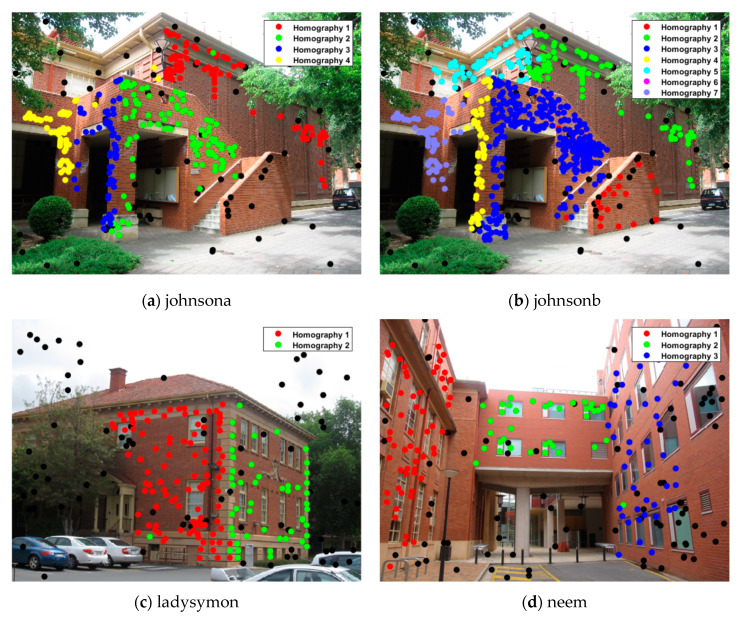

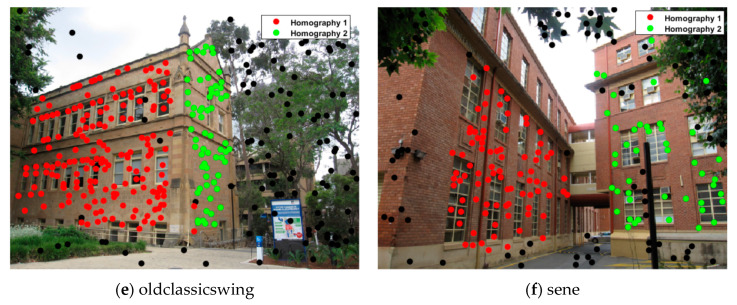
Inlier segmentation results for the multi-homography estimation.

Table 1 shows the misclassification results of the state-of-the-art methods and the proposed method for multi-homography estimation, where the results of the other four methods were obtained from [22]. It can be seen that the proposed method obtains the lowest misclassification result on most of the data sets and obtains the third lowest rate on “oldclassicswing”, which is actually very close to the lowest rate. The corresponding inlier segmentation preference results are presented in Figure 7, in which the black dots represent the outliers detected in Section 3.1 and most of the inliers for the different homography models can be segmented quite accurately. For the “johnsonb” data set, inliers for all four models are segmented accurately, which occupy the majority of all the points, but the inliers for models with a smaller inlier scale are easily mixed up and poorly segmented.

As a matter of fact, most of the multi-model fitting methods do not work well when dealing with a data set such as “johnsonb”, which contains quite a lot of models with highly varied inlier scales. This results in the sampling being uneven and the sampling rate on models with a smaller inlier scale being too low for the preferences to present a consensus on the inliers. Although the sub-region random sampling method can overcome the uneven sampling problem, the improvement is quite limited without the consideration of the distribution of the model inliers, which is quite difficult to obtain without sampling and preference analysis or a priori knowledge. The alternative sampling and clustering framework make it possible to sample the inlier clusters, but the performance is greatly dependent on the initial inlier segmentation results.

### 4.2. Multi-Fundamental Matrix Estimation

**Figure 8:**
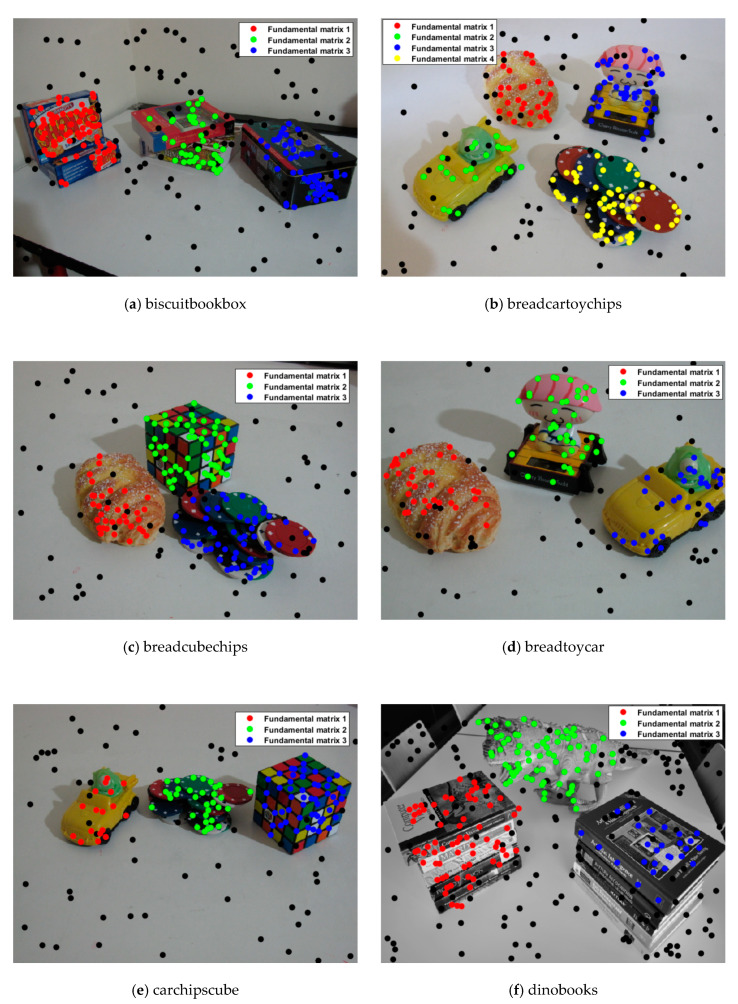
Inlier segmentation results for the multi-fundamental matrix estimation.

The misclassification results of the state-of-the-art methods and the proposed method for multi-fundamental estimation are presented in Table 2, where the results of the other four methods were obtained from [22]. The proposed method obtains the lowest misclassification error on all six experimental data sets, and even obtains zero misclassification error on three of them. The corresponding inlier segmentation preference results are presented in Figure 8, in which the black dots represent the outliers detected in Section 3.1 and most of the inliers for the different fundamental matrix models can be segmented quite accurately.

Overall, the proposed method works quite well in the multi-fundamental estimation experiment. For the “dinobooks” data set, the proportion of outliers (43%) is very high, and eight points need to be randomly selected every time to generate a fundamental matrix hypothesis, which results in the proportion of good hypotheses being very low and seriously impacts the performance of the preferences.

### 4.3. Outliers Detection

**Figure 9:**
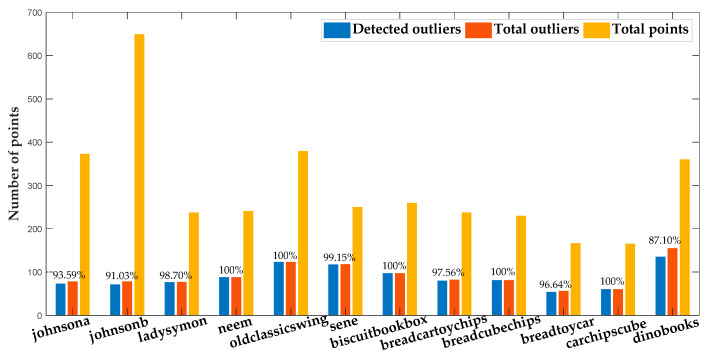
Outlier detection results on individual sequence.

The outlier detection results are presented in Figure 9, in which the outliers detected from all 12 data sets for the multi-homography estimation and multi-fundamental matrix estimation are shown. The ratio of the number of detected outliers to the number of total outliers is also listed in Figure 9. As can be seen from the outlier detection results, most of the outliers can be detected and with no inliers mixed over all the data sets and all the ratios of detected outliers are higher than 87%.

**Figure 10:**
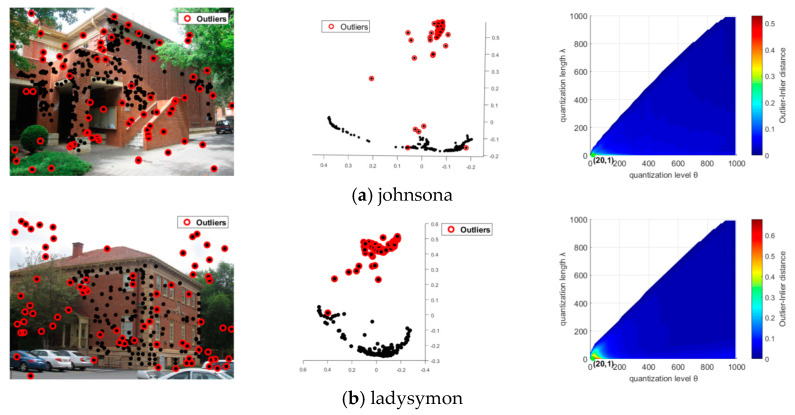

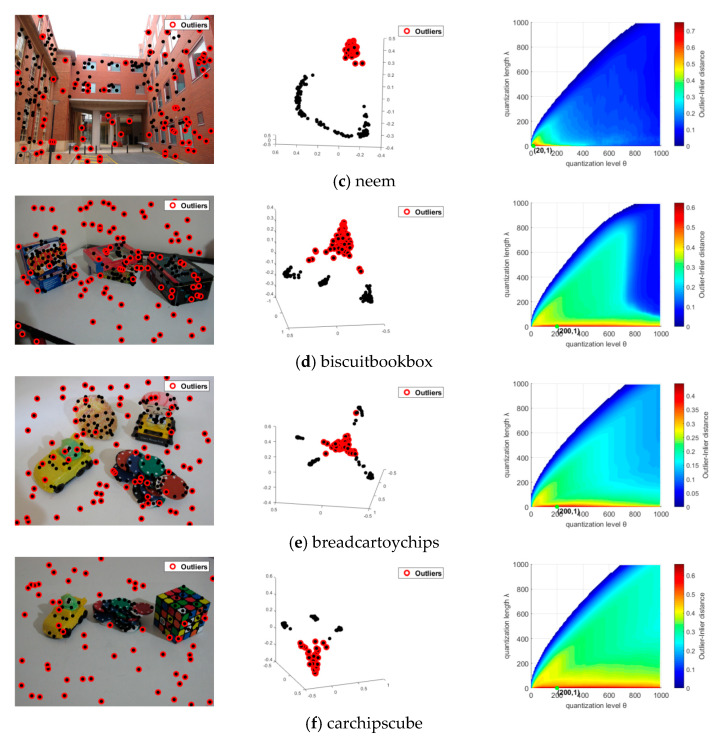
The impact of the quantization level and quantization length on the outlier detection.

During the outlier detection process, the key parameters are quantization level θ and quantization length λ, which have a great impact on the residual histogram preference for detecting outliers. In our experiments, these two parameters were set to be same for all six data sets in the multi-homography matrix estimation and multi-fundamental matrix estimation, respectively. In order to better distinguish inliers and outliers, the quantization length λ should set to a small value, which makes the residual histogram preferences of the inliers contain more nonzero elements, while the outliers will contain few non-zero elements. In the multi-homography matrix estimation, we set θ=20 and λ=1 for all six data sets. In the multi-fundamental matrix estimation, the model becomes more complicated, and θ=200 gave a good result in our experiments. In this paper, we define an outlier-inlier distance (OID) to measure the difference between the inlier and the outlier, and use OID in analyzing the parameter settings. We calculated the outlier-inlier distance (Equation (12)) for every combination of θ=1,10,20,⋯,1000, and λ=1,5,10,⋯,θ, and the results for all the data sets are presented in Figure 10.

Figure 10 shows the impact of the quantization level and quantization length on the outlier-inlier distance (OID in Equation (12)). The left column shows the outlier points in the images. The middle column shows the MDS plots for the data points in quantified residual preference space, where the quantization level θ=20 and the quantization length λ=1 for the multi-homography matrix estimation data (Figure 10a–c) and θ=200 and λ=20 for the multi-fundamental matrix estimation data (Figure 10d–f). The right column presents the *OID* change with quantization level θ and quantization length λ, in which areas with a color closer to red refer to bigger outlier-inlier distances and outliers that are more easily separated from the inliers.

It is clear that the change trends of *OID* are very close for the three multi-homography matrix data sets, which are also similar for the three multi-fundamental matrix data sets. The biggest OIDs for the multi-homography matrix data sets are near to the side where θ=20 and λ=1. Actually, θ∈[10,30] obtains a good detection result, and λ at the minimum value was applied for all the data sets in our experiments. Meanwhile, for the multi-fundamental matrix data sets, the biggest *OID*s arise along the *x*-axis, which means that the value of λ needs to be small, and θ∈[1,1000]. However, in the multi-fundamental matrix estimation experiments, when θ is too small, the outliers will be too dispersed to cluster into one group and, in practice, θ∈[100,800] can give a good result, and λ∈[10,50] can still ensure the separability of the outliers. We define the outlier-inlier distance (OID) as follows:

(12) $$\begin{array}{l} OID=\frac{\sum_{x_{i}^{O}\in c^{O}}\psi (x_{i}^{O},\chi (x_{i}^{O},k))}{card\left\{C^{O}\right\}}\\ \psi (x_{i},x_{j})=\left\|P^{MDS}(x_{i})-P^{MDS}(x_{j})\right\| \end{array}$$

here $c^{O}$ and $c^{I}$ refer to the outlier set and inlier set, and $P^{MDS}(x_{i})$ means the three-dimensional coordinate of $x_{i}$ when projected in MDS space. Then $\psi (x_{i},x_{j})$ is the distance of point $x_{i}$ and $x_{j}$ in MDS space, $\chi (x_{i},k)$ refers the *k*-nearest inlier to point $x_{i}$ in MDS space, $\chi (x_{i},k)=x_{\tau_{k}}^{I}$, where $\psi (x_{i}x_{\tau_{1}}^{I})\leq \psi (x_{i}x_{\tau_{2}}^{I})\leq \cdots \leq \psi (x_{i}x_{\tau_{k}}^{I})\leq \cdots \leq \psi (x_{i}x_{\tau_{n}}^{I})$ is the ascending order of the distance from outlier $x_{i}$ to all inliers $C^{I}=[x_{\tau_{1}}^{I},x_{\tau_{2}}^{I},\cdots ,x_{\tau_{k}}^{I},\cdots ,x_{\tau_{n}}^{I}]$. Then OID is the average distance of the *k*-th nearest inlier distance for each outlier. In our experiments, the value of *k* was set to 20.

## 5. Conclusions

In this paper, a robust geometric multi-model fitting method has been proposed. Firstly, the outliers in the data set are detected by means of linkage clustering with residual histogram preferences and then the inlier segmentation process is carried out on the data with outliers removed through linkage clustering, but permutation preferences are used in this stage, which shows superior performance for data sets with outliers removed. In order to obtain a stable clustering result after the linkage clustering, an alternative sampling and clustering framework is proposed in both the outlier detection and inlier segmentation processes. The experimental results also show that the outlier detection method based on residual histogram preference can successfully detect most of the outliers in the data. Overall, the proposed model fitting method can separate the inliers for different models and outperforms the state-of-the-art methods in geometric multi-model fitting.

In the future, the accuracy of proposed solution can be further improved by combining some state-of-the-art methods such as those described in [52,53,54,55] to preprocess the data. Then we perform model fitting based on the semantic information or classification results provided by these algorithms. The fitting results could be applied in solving the real-world motion segmentation problem, just as the work of [56]. Real image sequences data usually contain many outliers due to imperfections in data acquisition and preprocessing. The proposed geometric model fitting method can remove outliers and segment different motion models more accurately. The most important research direction following this paper could be multimotion visual odometry [57] and simultaneous localization and mapping (SLAM) [58] in dynamic scenes, where motion segmentation is a key step for separating the pixels corresponding to the dynamic background and the moving objects.

## Figures and Tables

**Table 1:** Misclassification (%) for the multi-homography estimation.

Methods	PEARL	J-Linkage	T-Linkage	SA-RCM	Proposed
johnsona	4.02	5.07	4.02	5.90	2.41
johnsonb	18.18	18.33	18.33	17.95	6.16
ladysymon	5.49	9.25	5.06	7.17	2.11
neem	5.39	3.73	3.73	5.81	1.24
oldclassicswing	1.58	0.27	0.26	2.11	0.53
sene	0.80	0.84	0.40	0.80	0.40

**Table 2:** Misclassification (%) for the multi-fundamental matrix estimation.

Methods	PEARL	J-Linkage	T-Linkage	SA-RCM	Proposed
biscuitbookbox	4.25	1.55	1.54	7.40	0
breadcartoychips	5.91	11.26	3.37	4.81	0.84
breadcubechips	4.78	3.04	0.86	7.85	0
breadtoycar	6.63	5.49	4.21	3.82	0.60
carchipscube	11.82	4.27	1.81	11.75	0
dinobooks	14.72	17.11	9.44	8.03	6.94
